# Magnesium Sulfate and Cerebral Oxygen Saturation in Mild Traumatic Brain Injury: A Randomized, Double-Blind, Controlled Trial

**DOI:** 10.3390/jcm11123388

**Published:** 2022-06-13

**Authors:** Hye-Min Sohn, Hyoeun Ahn, Won-Seok Seo, In-Kyung Yi, Jun Yeong Park

**Affiliations:** 1 Department of Anesthesiology and Pain Medicine, Ajou University School of Medicine, Ajou University Hospital, 164, World Cup-Ro, Yeongtong-gu, Suwon 16499, Korea; 109417@aumc.ac.kr (H.A.); wonseok612@aumc.ac.kr (W.-S.S.); imnothin@naver.com (I.-K.Y.); 2 Department of Trauma Nursing, Ajou University School of Medicine, Ajou University Hospital, Suwon 16499, Korea; junye11@aumc.ac.kr

**Keywords:** magnesium, multiple trauma, spectroscopy, near-infrared, cerebral oxygen saturation, neuroprotection, traumatic brain injury, analgesia, opioid consumption

## Abstract

Perioperative cerebral hypoperfusion/ischemia is considered to play a pivotal role in the development of secondary traumatic brain injury (TBI). This prospective randomized, double-blind, controlled study investigated whether magnesium sulfate (MgSO_4_) infusion was associated with neuroprotection in maintaining regional cerebral oxygen saturation (rSO_2_) values in patients with mild TBI undergoing general anesthesia. Immediately after intubation, we randomly assigned patients with TBI to receive either intravenous MgSO_4_ (30 mg/kg for 10 min, followed by a continuous infusion of 15 mg/kg/h) or a placebo (saline) during surgery. We also implemented an intervention protocol for a sudden desaturation exceeding 20% of the initial baseline rSO_2_. The intraoperative rSO_2_ values were similar with respect to the median (left. 67% vs. 66%, respectively; *p* = 0.654), lowest, and highest rSO_2_ in both groups. The incidence (left 31.2% vs. 24.3%; *p* = 0.521) and duration (left 2.6% vs. 3.5%; *p* = 0.638) of cerebral desaturations (the relative decline in rSO_2_ < 80% of the baseline value) were also similar for both groups. Although the patients suffered serious traumatic injuries, all critical desaturation events were restored (100%) following stringent adherence to the intervention protocol. Intraoperative remifentanil consumption, postoperative pain intensity, and fentanyl consumption at 6 h were lower in the MgSO_4_ group (*p* = 0.024, 0.017, and 0.041, respectively) compared to the control group, whereas the satisfaction score was higher in the MgSO_4_ group (*p* = 0.007). The rSO_2_ did not respond to intraoperative MgSO_4_ in mild TBI. Nevertheless, MgSO_4_ helped the postoperative pain intensity, reduce the amount of intraoperative and postoperative analgesics administered, and heighten the satisfaction score.

## 1. Introduction

Traumatic brain injury (TBI) is characterized by a variety of pathophysiological changes that occur immediately after trauma [1,2]. Moreover, secondary insult can cause irreversible changes in the brain, resulting in persistent injury-related difficulties and disabilities, even in mild TBI [3]. Surgery and anesthesia may subject the injured brain to secondary injuries as a result of inadequate cerebral oxygenation, changes in cerebral blood flow (hypoperfusion and hyperperfusion), impaired cerebrovascular autoregulation, cerebral metabolic dysfunction, and increased intracranial pressure; thus, the perioperative period is particularly important in the course of TBI management [1,4]. Given the complexity and dynamics of these changes, rapid diagnosis and vigilant neuromonitoring form the core principles of TBI management [5,6].

Perioperative magnesium sulfate (MgSO_4_), an N-methyl-D-aspartate receptor antagonist, is known to decrease pain and/or anesthetic/analgesic use, though its exact mechanism of action is unknown [7,8,9]. Magnesium is known to participate in vasodilation, hemostasis, and blood–brain barrier preservation and may also function as a potential neuroprotectant in acute stroke and brain hemorrhage [10,11]. Although one Cochrane systematic review found no evidence to support the use of magnesium salts in patients with acute TBI [12], subsequent evidence has emerged that magnesium plays a central role in the pathophysiology of TBI. Magnesium can protect neurons from ischemic damage and support neuronal survival following TBI through diverse mechanisms, such as through a cofactor of cellular energy metabolism and protein synthesis, as a potent calcium channel blocker [13], and via involvement in the mitigation of the cellular changes owing to global ischemia during trauma, suppression of cortical spreading depression, and relaxation of vascular smooth muscle, thereby possibly increasing cerebral blood flow [14]. One animal head injury model demonstrated that hypomagnesemia is associated with poor neurologic outcomes and increased mortality [15], whereas restoring magnesium levels reduces brain edema and enhances neurological and cognitive outcomes [16]. A recent clinical study found that magnesium was associated with a lower increase in hematoma volume and improved patient outcomes after intracerebral hemorrhage [17].

Near-infrared spectroscopy (NIRS) is a continuous, non-invasive method that facilitates the measurement of the regional cerebral oxygen saturation (rSO_2_). NIRS has been shown to reflect the cerebral mixed venous oxygen saturation. It is a useful modality for monitoring the balance of cerebral oxygen supply and demand during surgery with a high risk of cerebrovascular complications [2,18]. As patients subjected to multiple traumas present with mild to severe TBIs, NIRS can be used to assess the cerebral oxygenation status, guide its optimization, and predict the prognosis of brain function in these patients.

We hypothesized that the intraoperative administration of MgSO_4_ may be related to the prevention of deleterious secondary events in patients with TBI, as evidenced by rSO_2_ value changes. We also implemented an intervention protocol to hinder or restore cerebral desaturation. Therefore, we conducted this prospective, randomized double-blinded study to measure the hitherto uninvestigated brain oxygenation/desaturation state using NIRS in TBI patients under general anesthesia, while simultaneously evaluating the neuroprotective effects of MgSO_4_. As secondary outcomes, we expected MgSO_4_ to alleviate the pain felt after multiple trauma surgery and reduce perioperative analgesic consumption.

## 2. Materials and Methods

### 2.1. Study

This study was approved by the institutional review board of Ajou University Hospital (IRB No. AJIRB-MED-CT4-19-377), Suwon, Korea, on 1 November 2019, and registered at cris.nih.go.kr (Date of registration 4 June 2020, Registration No. KCT0005091). All patients provided written informed consent before surgery.

### 2.2. Patients

Eighty patients who were admitted to a tertiary academic medical and level-1 trauma center were enrolled in this prospective randomized study. The inclusion criteria were as follows: patients who experienced TBI within the last month, undergoing traumatic orthopedic surgery under general anesthesia, receiving or not receiving supplemental oxygen due to various lung injuries in the ward or intensive care unit, aged between 20 and 70 years, with a BMI between 18 and 35 kg/m^2^, and with an American Society of Anesthesiologists physical status of I, II, or III. The exclusion criteria for the study were patients with end-stage renal failure, atrioventricular block, or neurological disorder; already intubated patients for whom extubation was not possible after surgery; those diagnosed with a psychiatric disorder or drug or alcohol addiction; patients with previous history of stroke or brain surgery; those who refused to participate in the study; and patients with cognitive impairments or any other physical or mental illness that rendered them unable to provide a pain score.

### 2.3. Anesthesia and Monitoring

No premedication was administered before the administration of general anesthesia. Standard monitoring was established using electrocardiography, non-invasive blood pressure measurement, peripheral oxygen saturation, and a bispectral index sensor (BIS, A-2000 BIS™ monitor; Aspect^®^ Medical Systems Inc., Norwood, MA, USA) before the induction of anesthesia. Bilateral NIRS sensors (INVOS 5100; Covidien, Dublin, Ireland) were placed above the eyebrow on either side of the forehead, i.e., left (rSO_2_ L) and right (rSO_2_ R), to monitor the rSO_2_. Baseline rSO_2_ was measured for more than 1 min in the supine position without any medication before anesthesia, and the measurement was continued until the patient was transferred to the intensive care unit. Some patients received supplemental oxygen through various methods, depending on their condition, and the baseline rSO_2_ values were measured while maintaining the oxygen supplementation during transfer from the intensive care unit to the operating room. The intraoperative fraction of inspired oxygen (FiO_2_) was maintained at 0.5, and the minimal and maximal values of rSO_2_ and the maximal degree of desaturation were also recorded.

Anesthesia was induced by a continuous infusion of remifentanil via a target-controlled infusion pump (Orchestra^®^, Fresenius vial, Brezins, France) (2–4 µg/mL) and propofol 1.5–2.5 mg/kg. Rocuronium 0.6 mg/kg was administered to facilitate tracheal intubation after ensuring loss of consciousness. After intubation, the patients were administered either MgSO_4_ or saline according to a randomization list given to the blinded anesthesiologist: the Mg group received MgSO_4_ 30 mg/kg intravenously for 10 min, followed by a continuous infusion of 15 mg/kg/h during surgery, whereas the control group received the same volume of isotonic saline. This bolus dose, which was 60% of that of previous studies [8,9], was intended to prevent potential hemodynamic instability induced by rapid administration of a high dose of magnesium to patients with acute and severe traumatic injuries. Thereafter, anesthesia was maintained with sevoflurane, whose concentration was adjusted depending on the BIS value. The target concentration of remifentanil was adjusted to maintain arterial pressure and heart rate within 20% of the preoperative values. Controlled ventilation was adjusted to an end-tidal CO_2_ level of 4.0–4.7 kPa.

We endeavored to prevent the incidence of hypotension (mean blood pressure > 65 mmHg), anemia (hemoglobin > 7 g/dL), hypoxemia (arterial partial pressure of oxygen >100 mmHg), and hypothermia (core temperature > 35.5 °C) during surgery. After the return of the fourth twitch of the train-of-four response, glycopyrrolate 0.01 mg/kg and neostigmine 0.03 mg/kg were administered. The patient was extubated in the operating room when the train-of-four ratio recovered to ≥0.90 and was transferred to the postanesthetic care unit or the trauma intensive care unit.

### 2.4. Intervention Protocol

For all patients using rSO_2_, we had a strict intervention protocol for the occurrence of desaturation. We ensured that there was no external compression of the INVOS-NIRS electrodes during recording. After establishing the baseline value for each side, rSO_2_ changes during the duration of anesthesia were recorded. There were no changes in the degree of head tilt or rotation during the procedure. A sudden fall in rSO_2_ value exceeding 20% of the initial baseline value was designated as critical. In the event of a critical decrease in rSO_2_, the neck position was first checked to eliminate the possibility of external compression. The second step involved increasing the blood pressure, inspired concentration of FiO_2_, and end-tidal CO_2_ to approximately 40 mmHg and optimization of the preload using a mini-fluid bolus challenge of 100 mL [18,19]. Finally, if all interventions failed to restore the rSO_2_ value above 80% of the baseline value, a transfusion of red blood cells was considered at hemoglobin levels of 8–9 g/dL.

### 2.5. Assessment of Outcomes

The primary outcome was cerebral oximetry measurements at baseline and throughout surgery. The frequency and duration of cerebral desaturation was also reviewed. Postoperative pain was evaluated by a blinded investigator, who questioned patients about their pain using a numeric rating scale, which ranged from 0 (free of pain) to 10 (worst pain imaginable), at 6, 24, and 48 h after surgery. The cumulative consumption of analgesics via patient-controlled analgesia and rescue analgesics was recorded at each time point. The incidence of postoperative nausea and vomiting, use of rescue antiemetics, and the patients’ overall satisfaction score (subjective assessment of the patient, numeric rating scale = 10, was indicative of “very satisfied”) were recorded by a blinded investigator. The preparation and administration of parenteral drugs and the collection and measurement of data were performed by doctors and nurses who were blinded to the study group.

### 2.6. Sample Size Calculation

The sample size was determined based on the results of a previous study [8]. Baseline rSO_2_ was 52.8 ± 11.5 (%) in cardiac surgery for the control group, and an increase of 15% in rSO_2_ was considered clinically significant. The calculated sample size was 70 with an alpha error of 0.05 and power of 80%. The required sample size was established to be 80 patients, accounting for a 15% attrition rate.

### 2.7. Statistical Analysis

The results are presented as absolute values, means (SD), frequencies (percentages), or medians (IQR) after assessing the normality of the distribution using the Kolmogorov–Smirnov test. Continuous data were compared using the Student’s *t*-test, Mann–Whitney U test, ANOVA, or the Kruskal–Wallis test with post hoc analysis, wherever appropriate. The incidence data were compared using the *X*^2^ test or Fisher’s exact test, according to the expected counts. The changes between the time points within each group were compared using repeated-measures ANOVA. All statistical analyses were performed using SPSS software (version 25.0; SPSS Inc., Chicago, IL, USA), and the significance level was set to 0.05 for all tests.

## 3. Results

**Figure 1:**
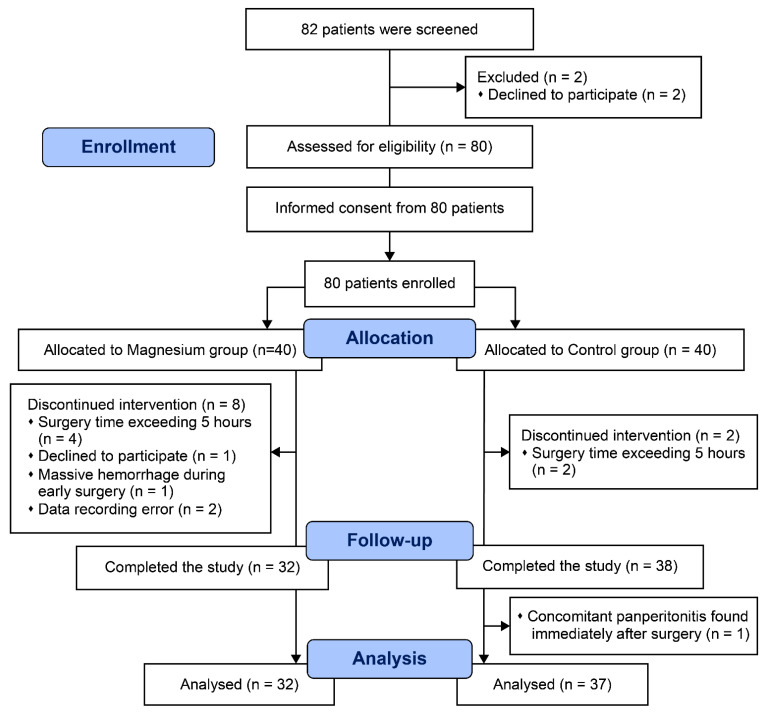
CONSORT diagram.

Eighty patients were initially assessed for eligibility for inclusion in this study. Eleven patients were excluded from the final analysis for the following reasons: one patient from the Mg group declined to participate; another from the Mg group experienced a massive bleeding event early during surgery; data recording errors occurred in two patients from the Mg group; one patient from the control group had concomitant panperitonitis requiring another urgent surgery; and the operative time exceeded 5 h in four and two patients from the Mg and control groups, respectively (Figure 1). The remaining 69 patients were included in the analysis.

The demographic and surgical factors of the 69 patients are provided in Table 1. Road traffic accidents were the cause of trauma in a large portion of the study population. The average interval between injury and current surgery was approximately 5 days in both groups. The preoperative magnesium levels at admission were similar for both groups.

The most common TBI symptoms included loss of consciousness, headache, and somnolence. The severity of TBI was mild in all cases (Table 2). Common findings on brain computed tomography were subarachnoid hemorrhage, subdural hemorrhage, and scalp swelling. In more than half of the patients, no evidence of trauma-related intracranial hemorrhage or skull fracture was found. The Glasgow Coma Scale score at the time of arrival to the trauma bay, exit from the trauma bay, and immediately before surgery (Mg group, 14.97 vs. control group, 14.92) were similar for both groups. On the contrary, the injury itself was severe in both groups according to Injury Severity Score (ISS, 21.2 vs. 22.0).

**Figure 2:**
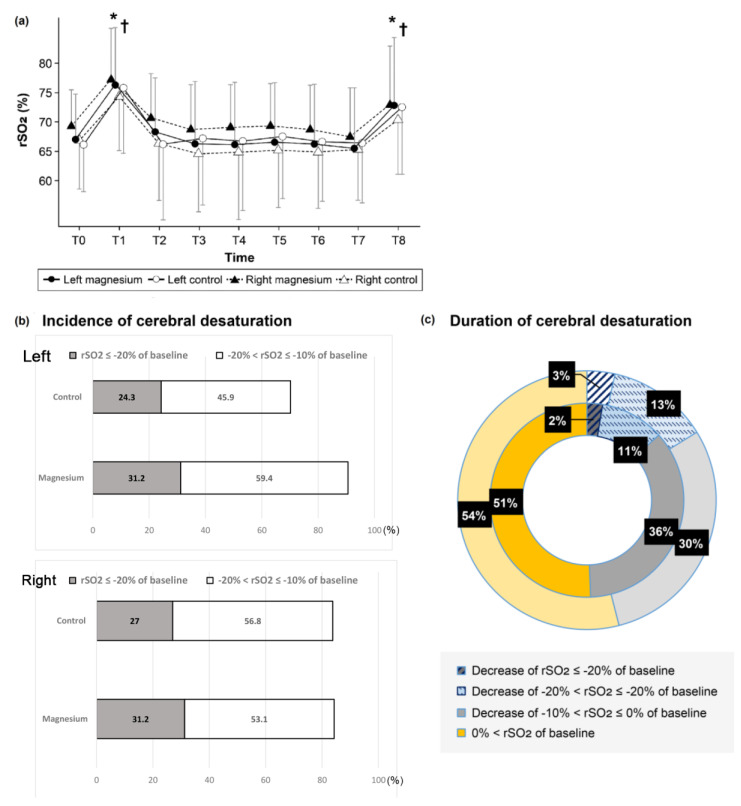
Representative values of regional cerebral oxygen saturation (rSO_2_) in the operating room. (**a**) Time trend of rSO_2_. T0: baseline at admission to operating room (OR), T1: Mg bolus administration, T2: Mg continuous infusion, T3–T6: 10, 20, 30, 40 min after Mg infusion, T7: end of surgery, T8: leaving the OR. ● Left ▲ Right ● Magnesium ○ control. (**b**) Incidence of percentage decreases in rSO_2_ (%), below or equal to minus 20% and below or equal to 10% to less than 20% relative to each baseline value. (**c**) Duration of percentage decreases in rSO_2_ (%). The inner circle is the Magnesium group. The outer circle is the Control group. * *p* < 0.05 from baseline value in Magnesium (Mg) group, ^†^ *p* < 0.05 from baseline value in control group.

Regarding the primary outcome measures, the cerebral oximetry measurements, including the baseline (left 67.0 ± 7.8% vs. 66.1 ± 8.0%; *p* = 0.654), lowest and highest intraoperative rSO_2_ values, were similar for both groups (Figure 2, online Supplementary Material Table S1). The absolute rSO_2_ values when FiO_2_ was elevated, which occurred at the time of magnesium bolus administration and at extubation, were found to be significantly greater than the baseline rSO_2_ values (Figure 2a). However, there was no difference between the baseline values and those obtained at other time points. The incidence of rSO_2_ < 20% (critical events) and that between 20% and 10% were similar in both groups (Figure 2b, left *p* = 0.521 and *p* = 0.265, respectively). The duration of these cerebral desaturation percentages did not differ between the groups (Figure 2c, left *p* = 0.638 and *p* = 0.675, respectively). In the subgroup analysis performed according to whether or not oxygen was administered, the rSO_2_ values were not different within each group. The critical desaturation events were reversible in all cases due to stringent adherence to the intervention protocol. Red blood cells were similarly transfused between the groups and independently of the rSO_2_ intervention protocol.

The amount of remifentanil infused during surgery was significantly lower in the Mg group (221.1 ± 148.4 mcg vs. 314.6 ± 181.9, *p* = 0.024) than in the control group. The magnesium level at the end of the surgery in the Mg group was higher, and its value approximated the upper limit of the normal range (2.68 ± 0.32 mg/dL vs. 1.95 ± 0.22 mg/dL, *p* = 0.000). The amount of phenylephrine, total duration of hospitalization, and physical activity at discharge were similar between the groups (online Supplementary Material Table S2).

The pain intensity was lower and satisfaction scores were higher in the Mg group during the first 6 h postoperatively than in the control group (*p* = 0.017 and 0.007, respectively) (Table 3). Cumulative fentanyl-equivalent consumption (including fentanyl patient-controlled analgesia, fentanyl rescue and tramadol) (191.6 ± 143.6 vs. 280.9 ± 201.6, *p* = 0.041) was lower in the Mg group 6 h postoperatively.

There was no difference between the frequency and dose of analgesics (fentanyl rescue, acetaminophen, tramadol, and nefopam) administered after surgery between the groups; however, the frequency of nonsteroidal anti-inflammatory drug use was lower in the Mg group at 6 h postoperatively. There was no difference in the incidence of postoperative nausea and vomiting and the use of rescue antiemetics between the groups (28% vs. 32%, *p* = 0.698). The initial and intraoperative hemodynamic variables did not differ between the groups (online Supplementary Material Table S3). No magnesium-related untoward side effects, such as bradycardia, electrocardiographic changes, respiratory depression, delayed reversal of the neuromuscular blockade, or delayed discharge from the post-anesthesia care unit, were reported.

## 4. Discussion

This prospective randomized, double-blind controlled study investigated the relationship between the intraoperative administration of MgSO_4_ and the changes in the rSO_2_ values in patients with TBI. It revealed no association in both the *absolute* rSO_2_ values and the incidence and duration of the *relative* decrease in the rSO_2_ values < 80% of baseline. After strict compliance to our intervention protocol, critical desaturation events in all cases were restored. Remifentanil consumption was significantly lower in the Mg group than in the control group. The pain intensity and fentanyl consumption were lower during the first 6 h postoperatively, and the satisfaction score was higher in the Mg group.

Preventive measures addressing brain protection in TBI deserve a high priority to alleviate additional harm. Subsequent injuries can be incurred, especially when patients with TBI are required to undergo major surgery under general anesthesia as a result of hypotension, hypoxemia, hypo- or hypercarbia, fever, and hypo- or hyperglycemia [1,4]. This secondary brain injury contributes to the increase in healthcare costs, prolonged hospitalization, poor functional outcomes, increased postoperative complications, and even mortality [3,20].

Although the exact pathophysiology of secondary TBI is unclear, decreased cerebral perfusion and oxygenation are closely related mechanisms [1]. Cerebral oxygenation is well known as a potentially modifiable risk factor for TBI, and if insufficient cerebral oxygenation can be accurately and timely detected, it can be improved through adaptation/correction of the relevant variables [5,19,21]. Therefore, NIRS can be a practical option for perioperative rSO_2_ monitoring, as it provides an opportunity for the early detection of cerebral oxygen supply/demand imbalance. Recent studies detailed the potential therapeutic roles of MgSO_4_ in vasodilation, hemostasis, blood–brain barrier preservation, and direct neuroprotection [12,22,23]. Hypomagnesemia in TBI is associated with poorer outcomes [15,24]. MgSO_4_ can reliably confer cerebroprotective effects in animal models of TBI [11,15,25], though clinical studies have not shown consistent beneficial effects. Therefore, the administration of magnesium to patients with TBI could theoretically help mitigate secondary injury.

However, contrary to our expectations, no significant association was observed between the intraoperative changes in rSO_2_ and magnesium infusion in the current study. The absolute degree of decline was similar in the two groups. Comparison of the relative percentage decrease, which has greater clinical utility, also revealed that the incidence and duration of rSO_2_ reduction below 80% of the baseline was not affected by magnesium administration.

The potential causes for the current observation are as follows. First, our participants had mild TBI; consequently, we found that the rSO_2_ levels did not fluctuate rapidly in mild TBI as long as adequate brain tissue oxygenation and vascular hemostasis could be maintained. We may expect different results if similar studies are conducted in patients with moderate or severe TBI. Our initial rSO_2_ values were higher than those in patients undergoing cardiac surgery with cardiopulmonary bypass, who have been studied extensively in this regard. We observed desaturations greater than 10% from baseline in 50% of patients, and greater than 20% from baseline in 30% of patients. In one study on cardiac surgery, corresponding decrements occurred in 60% and 40% of patients, respectively [21]. The absolute baseline, maximal, and minimal values were also 8–10% higher in patients with mild TBI than in patients undergoing cardiac surgery (online Supplementary Material Table S1) [26]. Contrary to the severity of the patients’ injury (ISS 21.2 vs. 22.0, an ISS of 16–24 is considered severe, 25 and higher very severe), few patients showed markedly low levels of baseline rSO_2_. Additionally, the predictive or diagnostic role of NIRS in monitoring the progression or mitigation of TBI may not be relevant in the absence of significant hemodynamic disturbances, i.e., in non-pulsatile perfusion.

Second, NIRS monitoring itself could have influenced the favorable outcomes in this study. NIRS monitoring alone has been shown to be associated with the amelioration of secondary brain injury [2,27]. We prospectively implemented an intervention protocol to reverse cerebral desaturation in both groups [19,28], which prevented a potential deleterious situation very early. Monitoring and applying the mandatory corrective intervention protocol in our institution were effective in restoring cerebral oxygenation in all patients, both of which could play powerful neuroprotective roles in this study. Third, the cerebral oxygen saturation measured by NIRS does not reflect the cerebral oxygen utilization or that cerebral hypoxia is not the principal driver of the TBI course. The influence of the oxygenation of the extracerebral tissue cannot be excluded due to the technical limitations of the non-invasive monitoring device [18]. Moreover, it is not possible to monitor multiple brain regions using NIRS.

Fourth, the dose or duration of magnesium infusion could have been insufficient to alter the course of TBI [9,14]. An adequate magnesium level at admission is an indicator of neuroprotection [10,17], and our initial magnesium levels were not low at the time of admission and immediately after surgery, even in the control group. While the dose of magnesium required to reduce pain and anesthetic usage has been studied extensively (30–50 mg/kg bolus), the respective doses required to improve microcirculation (through vasodilation of the cerebral vascular beds) and reduce cerebral edema (by maintaining and preserving blood–brain barrier permeability) need further investigation [29]. Fifth, the incidence of hypotensive events was similar between the groups, which were appropriately handled according to our management protocol. Abrupt changes in cerebral perfusion, especially reperfusion following a hypoperfusion event, are considered to be factors associated with additional brain injury [30]. There was no association between rSO_2_ levels falling below 80% of the baseline and hypotensive events in the current study. This indicates that the transient changes in cerebral perfusion cannot be accurately reflected by rSO_2_ monitoring and highlights the importance of strict blood pressure management during surgery in patients with TBI.

The intensity of pain was substantially stronger in patients with multiple traumas in the present study, than in those who underwent a single orthopedic surgery [31]. Particularly, the patients identified very severe pain (8.2 in the numeric rating scale) at 6 h postoperatively in the control group, despite receiving patient-controlled analgesia and injections of opioid and non-opioid rescue analgesics. Moderate pain continued in both groups until 24 h postoperatively. Consistent with our results, conventional opioid-based pain protocols in surgical patients often remain suboptimal for pain control [32]. Furthermore, patients undergoing major trauma surgery already experience pre-existing concurrent pain, including in other parts of the body, making it more difficult to manage the acute pain added with the current surgery [33]. Recently, multimodal pain management integrates the use of several analgesic medications targeting a different pain-related receptor to maximize pain relief and minimize adverse effects [34]. The mechanisms underlying the antinociceptive effects of MgSO_4_ include the inhibition of calcium influx, antagonism of N-methyl-D-aspartate (NMDA) receptors, and inhibition of enhanced ligand-induced NMDA signaling in hypomagnesemia. This calcium channel blocker effect augments opioid-induced analgesia, decreases total opioid consumption, and even attenuates central sensitization or delays the development of opioid tolerance [7,35]. Thus, we demonstrated the representative advantages of intraoperative MgSO_4_, which ameliorates pain and is capable of reducing analgesic requirements, which remain consistent with many other previous studies [8,31]. Patient satisfaction increased with the gradual decrease in pain.

The present study is subject to the following limitations. First, baseline rSO_2_ was measured when the patient entered the operating room, regardless of supplemental oxygen to some patients. Patients who experienced multiple traumas may receive oxygen depending on their condition. The oxygenation state and initial rSO_2_ levels at the time of entering the operating room are known to be associated with the prognosis regardless of oxygen supplementation [36]. The rSO_2_ does not seem to deteriorate substantially if adequate oxygen supply and proper management are performed, even in patients with hemodynamic instability or respiratory distress. Second, we did not extend the rSO_2_ measurement to the immediate postoperative period or intensive care unit stay, which might also be a potential confounder of the observed results and subsequent prognosis. Third, we did not assess cerebral injury using different parameters such as neurocognitive function and brain injury serum biomarker release (e.g., S100B), which could have represented positive differences between the groups. Invasive monitoring, such as intracranial pressure monitoring, was not used for the comparison of the effect of MgSO_4_. Fourth, outpatient follow-up was performed selectively only in a few patients because of the low severity of TBI. Therefore, comparisons of postoperative functional outcomes, such as the Glasgow Outcome Score and modified Rankin scale, were not possible between the groups.

This is the first study to investigate the relationship between MgSO_4_ infusion and rSO_2_ in TBI, exploring the neuroprotective effect of MgSO_4_ on TBI with respect to its safety and potential efficacy. Together, we expanded the applicability of NIRS and determined the incidence and extent of cerebral desaturation in patients with TBI who were susceptible to cerebral hypoxia. However, it was difficult to envision the brain environment or predict outcomes via NIRS when there was substantial regional heterogeneity. Instead, we clearly obtained the benefits of NIRS-based corrective interventions by sequentially restoring cerebral desaturation and reaffirmed the analgesia-potentiating and opioid-sparing effects of MgSO_4_.

## 5. Conclusions

In summary, continuous perioperative rSO_2_ monitoring with NIRS did not demonstrate the diagnostic or clinical benefit of MgSO_4_ in patients with mild TBI undergoing major orthopedic surgery. We suggest evaluating the role of rSO_2_ monitoring in patients with various TBIs at risk of neurologic complications and assessing the benefits of NIRS-based corrective interventions. Additionally, further studies are needed to extend our findings of MgSO_4_ to cases of more severe TBI, to determine the effects of various doses and durations of MgSO_4_ aimed at optimizing cerebral oxygenation and intracranial physiology, and to prevent or mitigate secondary injury. Even if negative results are obtained, as in the present study, future research will be able to identify the benefits of reducing analgesic consumption and pain relief through MgSO_4_ administration.

## Figures and Tables

**Table 1:** Baseline characteristics of patients and perioperative data assigned to magnesium or the control group.

	Magnesium Group (*n* = 32)	Control Group (*n* = 37)	*p* Value
Sex (Male/Female)	25/7	32/5	0.361
Age (year)	49.5 ± 15.2	50.1 ± 14.9	0.879
Height (cm)	168.5 ± 7.2	167.9 ± 8.1	0.749
Weight (kg)	71.9 ± 11.6	69.9 ± 11.4	0.479
ASA (I/II/III)	3/18/11	3/28/6	0.211
Mechanism of Injury, *n* (%)			0.642
Fall	11 (34.4%)	11 (29.7%)	
Transportation accident	20 (62.5%)	26 (70.3%)	
Explosion	1 (3.1%)	0 (0%)	
Days after injury (days) (range)	4.5 ± 3.7 (6 h–13 day)	5.1 ± 4.7 (6 h–21 day)	0.569
Surgery, Orthopedic			0.315
Upper extremity	11 (34.4%)	9 (24.3%)	
Lower extremity	12 (37.5%)	15 (40.5%)	
Hip	4 (12.5%)	4 (10.8%)	
Other parts	4 (12.5%)	6 (16.2%)	
Combined op	1 (3.1%)	3 (8.1%)	
Magnesium at admission (normal range: 1.6–2.6) (mg/dL)	1.96 ± 0.41	2.03 ± 0.20	0.361
Duration of surgery (min)	81.1 ± 43.0	99.6 ± 63.3	0.167
Duration of anesthesia (min)	126.4 ± 49.0	143.1 ± 66.7	0.247
Aldrete score *	10 (*n* = 20)	10 (*n* = 25)	
PACU stay time (min) *	34.6 ± 7.7 (*n* = 20)	37.8 ± 9.9 (*n* = 25)	0.239

ASA, American Society of Anesthesiologists; PACU, post-anesthesia care unit. Values are number (proportion) or mean ± SD. * only those who have been transferred to PACU after surgery.

**Table 2:** Traumatic brain injury aspects preoperatively.

	Magnesium Group (*n* = 32)	Control Group (*n* = 37)	*p* Value
TBI, Symptom, *n* (%)			
Loss of consciousness at the time of injury	24 (75.0%)	33 (89.2%)	0.121
Headache	6 (18.8%)	8 (21.6%)	0.767
Dizziness	3 (9.4%)	6 (16.2%)	0.489
Nausea/vomiting	5 (15.6%)	5 (13.5%)	0.804
Memory impairment	5 (15.6%)	6 (16.2%)	0.947
Sleeping tendency	10 (31.3%)	13 (35.1%)	0.733
TBI, Severity, *n* (%) immediately before surgery			
Mild (GCS 15/14)	31/1	34/3	0.618
Moderate	0	0	
Severe	0	0	
Brain CT, *n* (%) *			
Subarachnoid hemorrhage	8 (25.0%)	5 (13.5%)	0.224
Subdural hemorrhage	6 (18.8%)	4 (10.8%)	0.350
Epidural hemorrhage	4 (12.5%)	2 (5.4%)	0.349
Intracerebral hemorrhage	0 (0%)	1 (2.7%)	0.279
Cerebral Contusion	2 (6.3%)	4 (10.8%)	0.503
Skull fracture	7 (21.9%)	3 (8.1%)	0.105
Scalp/soft tissue swelling	8 (25.0%)	14 (37.8%)	0.254
Midline shift > 5 mm, *n* (%)	0	0	
The others	4 (12.5%)	3 (8.1%)	0.547
No intracranial hemorrhage or bony skull fracture	16 (50.5%)	17 (51.5%)	0.737
TBI site (Left/Right/Both/non-specific/none)	(3/9/4/0/16)	(4/4/4/8/17)	
Preoperative antiepileptic prescription	10 (31.3%)	6 (16.2%)	0.140
GCS at the time of entering trauma-bay	14.16 ± 2.02	14.62 ± 0.83	0.210
15/14/13–11/less than 11, *n*	24/6/1/1	21/11/4/1	
GCS at the time of leaving trauma-bay	14.62 ± 0.73	14.74 ± 0.56	0.451
15/14/13–11/less than 11, *n*	26/3/0/3	23/8/4/2	
Invasive ICP monitoring, *n*	0	0	
Evacuation of brain mass lesion, *n*	0	0	
ISS score			
Median (range, IQR)	22 (5–43, 17–23)	19 (9–43, 17–22)	
Mean ± SD	21.2 ± 6.7	22.0 ± 9.3	0.691

TBI, traumatic brain injury; GCS, Glasgow Coma Scale; CT, computed tomography; ICP, intracranial pressure; ISS, injury severity score; IQR, interquartile range. Values are number (proportion) or mean ± SD. * Allow duplicate counts because of concurrent brain injuries identified by CT scan.

**Table 3:** Postoperative analgesic consumption and pain scores during the first 48 h after surgery.

	Magnesium Group (*n* = 32)	Control Group (*n* = 37)	*p* Value
PCA fentanyl consumption	*n* = 17 (53.1%)	*n* = 24 (64.9%)	0.322
postoperative 6 h (mcg)	201.9 ± 134.1 *	302.8 ± 140.4	0.026
postoperative 24 h (mcg)	574.7 ± 380.7	598.6 ± 264.9	0.814
postoperative 48 h (mcg)	762.8 ± 464.4	853.8 ± 357.4	0.482
Fentanyl bolus iv until 6 h postoperatively (mcg)	*n* = 12 (37.5%) 75.0 ± 26.1	*n* = 11 (29.7%) 95.8 ± 54.2	0.495 0.243
Nefopam consumption	*n* = 23 (71.9%)	*n* = 31 (83.8%)	0.232
postoperative 6 h (mg)	21.6 ± 2.3	21.9 ± 2.5	0.700
postoperative 24 h (mg)	70.6 ± 8.5	71.9 ± 5.4	0.519
postoperative 48 h (mg)	86.2 ± 23.6	93.4 ± 8.1	0.137
NSAIDs use, number (%)			
postoperative 6 h	5 (15.6%) *	14 (37.8%)	0.039
postoperative 24 h	6 (18.8%)	8 (21.6%)	0.767
postoperative 48 h	8 (25.0%)	6 (16.2%)	0.366
Tramadol consumption	*n* = 29 (90.6%)	*n* = 36 (97.3%)	0.330
postoperative 6 h (mg)	56.3 ± 50.4	49.3 ± 47.3	0.558
postoperative 24 h (mg)	120.3 ± 71.9	104.1 ± 76.7	0.370
postoperative 48 h (mg)	116.4 ± 65.6	127.0 ± 74.9	0.536
Pain scores (NRS)			
postoperative 6 h	6.8 ± 2.8 *	8.2 ± 2.0	0.017
postoperative 24 h	5.1 ± 2.5	5.4 ± 2.3	0.660
postoperative 48 h	3.3 ± 2.2	3.2 ± 2.6	0.839
Satisfaction scores (NRS)			
postoperative 6 h	66.1 ± 28.9 *	47.1 ± 27.6	0.007
postoperative 24 h	71.8 ± 21.1	65.7 ± 20.6	0.226
postoperative 48 h	78.3 ± 21.3	73.4 ± 24.6	0.389

PCA, patient-controlled analgesia; NSAIDs, nonsteroidal anti-inflammatory drugs; NRS, numeric rating scale. Values are mean ± SD. * *p* < 0.05 = between groups.

## Data Availability

Data are available from the authors (HMS) upon reasonable request. The clinical data are fully anonymized, and the experiments were performed by a qualified anesthesiologist with good clinical practice in accordance with the relevant guidelines and regulations.

## Supplementary Materials

The following supporting information can be downloaded at: https://www.mdpi.com/article/10.3390/jcm11123388/s1. Table S1. Baseline and intraoperative regional cerebral oxygen saturation (rSO2) data. Table S2. Intraoperative anesthetic and postoperative clinical variables. Table S3. Perioperative hemodynamics and laboratory variables immediate after surgery.
