# Supplementary material for: Magnesium Sulfate and Cerebral Oxygen Saturation in Mild Traumatic Brain Injury: A Randomized, Double-Blind, Controlled Trial
Source: J Clin Med. 2022 Jun 13;11(12):3388. doi: 10.3390/jcm11123388 (PMC9225065; doi:10.3390/jcm11123388)
Supplement: Supplementary file 1 [file jcm-11-03388-s001.zip › jcm-1720581-SI.pdf]

Supporting Information

Table S1 Baseline and intraoperative regional cerebral oxygen saturation (rSO_2) data.

Table S2 Intraoperative anaesthetic and postoperative clinical variables.

Table S3 Perioperative hemodynamics and laboratory variables immediate after surgery.

Table S1 Baseline and intraoperative regional cerebral oxygen saturation (rSO_2) data.

**Cerebral Saturation Data**	**Magnesium group (n=32)**	**Control group (n=37)**	**P value**
**Left**			
Baseline rSO_2 at admission to OR (%)	67.0 ± 7.8	66.1 ± 8.0	0.654
Lowest rSO_2 (%)	57.6 ± 12.3	57.7 ± 11.5	0.985
Highest rSO_2 (%)	81.8 ± 8.2	81.2 ± 9.8	0.767
**Right**			
Baseline rSO_2 at admission to OR (%)	57.6 ± 12.3	57.7 ± 11.5	0.985
Lowest rSO_2 (%)	57.1 ± 11.9	54.3 ± 10.8	0.320
Highest rSO_2 (%)	81.4 ± 8.5	80.1 ± 9.7	0.558

OR, operating room.

Values are mean ± SD.

Table S2 Intraoperative anaesthetic and postoperative clinical variables.

	Magnesium group (n=32)	Control group (n=37)	P value
Crystalloids (ml)	592.8 ± 561.1	712.2 ± 508.2	0.357
Colloids (ml) intraoperatively	0	0	
Estimated blood loss (ml)	119.4 ± 112.8	164.3 ± 227.5	0.314
Transfused packed red blood cells, n (%) (units)	3 (9.4%) (1.3)	6 (16.2%) (1.2)	0.400
Urine (ml)	158.9 ± 214.7	174.6 ± 275.5	0.795
Propofol, induction (mg)	127.5 ± 19.2	123.2 ± 19.0	0.359
Remifentanil, total (mcg)	221.1 ± 148.4	314.6 ± 181.9	0.024
Rocuronium induction (mg)	47.7 ± 4.6	48.4 ± 3.7	0.473
Rocuronium added dose, n (%)	8 (25%), 4.7 ± 10.9	13 (30.4%), 8.2 ± 13.5	0.238
Ephedrine (mg)	(n=2) 0.5 ± 2.0	(n=3) 0.4 ± 1.6	0.875
Phenylephrine (mcg)	(n=11) 35.0 ± 66.2	(n=10) 26.2 ± 50.6	0.535
Magnesium at admission preoperative	1.96 ± 0.41	2.03 ± 0.20	0.361
Magnesium at the end of surgery ($mg.dl^{-1}$) (normal range: 1.6–2.6)	2.68 ± 0.32	1.95 ± 0.22	0.000
Admitted to ICU after this surgery, n (%)	12 (37.5%)	12 (32.4%)	0.659
Median days LOS (range, IQR)**	13.5 (4–35, 11–19)	13 (8–35, 11–20)	
In-hospital case-fatality rate, n (%)	0	0	
**Discharge destination, n (%)**			
Home	11 (34.4%)	6 (16.2%)	0.081
Other hospital or Rehabilitation	21 (65.6%)	31 (83.8%)	
**Level of activity at discharge**			
Ambulation	17 (53.1%)	13 (35.1%)	0.065
Wheelchair accessible	12 (37.5%)	15 (40.5%)	
Need bed rest	3 (9.4%)	9 (24.3%)	

LOS, length of stay.

Values are mean ± SD.

Table S3 Perioperative hemodynamics and laboratory variables immediate after surgery.

	**Magnesium group (n=32)**	**Control group (n=37)**	**P value**
Initial SpO_2 (%)	98.3 ± 2.7	97.5 ± 4.4	0.377
Initial SBP (mmHg)	142.3 ± 27.7	145.3 ± 23.9	0.630
Mean SBP (mmHg)	121.6 ± 17.1	121.4 ± 13.6	0.948
Initial DBP (mmHg)	74.7 ± 12.0	75.7 ± 14.2	0.757
Mean DBP (mmHg)	70.9 ± 10.3	68.8 ± 9.4	0.372
Initial MBP (mmHg)	89.9 ± 21.0	92.8 ± 14.5	0.505
Mean MBP (mmHg)	84.0 ± 11.2	83.7 ± 9.7	0.899
Initial HR ($beats.minute^{-1}$)	89.2 ± 24.0	89.1 ± 18.8	0.993
Mean HR ($beats.minute^{-1}$)	86.9 ± 13.1	82.9 ± 13.9	0.215
Mean $EtCO_2$ (mmHg)	32.6 ± 2.5	32.4 ± 2.8	0.746
Hematocrit (%) preoperative	34.5 ± 6.8	32.2 ± 7.2	0.189
Hematocrit (%) postoperative	34.5 ± 7.2	32.6 ± 5.2	0.231
Calcium total ($mg.dl^{-1}$) postoperative	8.35 ± 0.62	8.33 ± 0.62	0.891
Lactic acid ($mmol.l^{-1}$) postoperative	1.97 ± 1.19	2.20 ± 1.01	0.432
pH	7.39 ± 0.46	7.41 ± 0.06	0.199
pO_2 (mmHg)	149.3 ± 40.5	144.7 ± 38.8	0.749
pCO_2 (mmHg)	37.3 ± 4.5	35.7 ± 6.4	0.413
HCO_3 ($mmol.l^{-1}$)	22.8 ± 1.8	22.9 ± 2.2	0.937

SBP, systolic blood pressure; DBP, diastolic blood pressure; MBP, mean blood pressure; HR, heart rate.

Values are mean ± SD.
